# Nurse autonomy expressed in Portuguese and Brazilian professional legislation: a documentary study (1986–2022)

**DOI:** 10.1590/1980-220X-REEUSP-2023-0199en

**Published:** 2024-02-19

**Authors:** Maria Ligia dos Reis Bellaguarda, Paulo Joaquim Pina Queirós

**Affiliations:** 1 Universidade Federal de Santa Catarina, Florianópolis, SC, Brazil.; 2 Escola Superior de Enfermagem de Coimbra, Coimbra, Portugal.

**Keywords:** Professional Autonomy, Nursing, History, Professional Review Organizations, Sociology, Autonomía Profesional, Enfermería, Historia, Organizaciones de Normalización Profesional, Sociología, Autonomia Profissional, Enfermagem, História, Organizações de Normalização Profissional, Sociologia

## Abstract

**Objective:**

To analyze the convergence of nurse’s autonomy expressed in Brazilian and Portuguese professional practice legislation.

**Method:**

Qualitative, social-historical documentary study on the normalization of Brazilian and Portuguese professional standards for nursing practice, materials socialized in the digital collection of the profession’s organizational and disciplinary entities. Qualitative analysis from the perspective of Eliot Freidson’s sociology of professions.

**Results:**

Ten standards were analyzed, five from each country, which establish legislation for the nurses’ professional practice. The following categories emerged: autonomy of knowledge and specific competence of the profession, in the ethical limits of the multi-professional relationship and in the disciplining of training for professional practice.

**Conclusion:**

The professional autonomy under analysis implies providing access to services and to multi-professionality for the availability of health to society.

## RESUMO

**Objetivo:**

Analisar a convergência da autonomia da enfermeira-enfermeiro expressa na legislação do exercício profissional brasileiro e português.

**Método:**

Estudo qualitativo, sócio-histórico documental na normalização de padrões profissionais brasileiro e português do exercício da enfermagem, materiais socializados no acervo digital das entidades organizativas e disciplinares da profissão. Análise qualitativa sob a perspectiva da sociologia das profissões de Eliot Freidson.

**Resultados:**

Analisadas 10 normalizações, cinco de cada país, em que estabelecem as legislações para o exercício profissional da enfermeira-enfermeiro. Emergiram as categorias: autonomia de conhecimento e competência específica da profissão, na eticidade dos limites da relação multiprofissional e no disciplinamento da formação ao exercício profissional.

**Conclusão:**

A autonomia profissional em análise implica em oportunizar o acesso aos serviços, à multiprofissionalidade para a disponibilidade de saúde à sociedade.

## RESUMEN

**Objetivo:**

Analizar la convergencia de la autonomía enfermera-enfermero expresada en la legislación de práctica profesional brasileña y portuguesa.

**Método:**

Estudio documental cualitativo, sociohistórico, sobre la normalización de los estándares profesionales brasileños y portugueses para la práctica de enfermería, materiales socializados en el acervo digital de las entidades organizativas y disciplinarias de la profesión. Análisis cualitativo desde la perspectiva de la sociología de las profesiones de Eliot Freidson.

**Resultados:**

Se analizaron 10 normas, cinco de cada país, que establecen la legislación para el ejercicio profesional de la enfermería. Surgieron las categorías: autonomía del conocimiento y competencia específica de la profesión, en los límites éticos de la relación multiprofesional y en la disciplina de la formación para el ejercicio profesional.

**Conclusión:**

La autonomía profesional bajo análisis implica posibilitar acceso a los servicios, multiprofesionalidad para la disponibilidad de la salud a la sociedad.

## INTRODUCTION

The limits of professional autonomy exist for any and all occupations, and they depend on the working condition, history in a professional organization, which reiterates the importance of ethics, self-regulation, and formal education.

Issues within the scope of professional autonomy permeate the level of competence, transposition of ideas for assistance with a view to configuring independence. Therefore, within the scope of the Nursing profession, the autonomy of some points of view must be analyzed and, within the social-historical scenario, seek a framework that strengthens sociological discussions, from care practices to teaching and research, the evolution or involution, which occur within the logic of professional organization. This corroborates the complexity in expanding the knowledge of nurses, to resolve and meet social needs^(1)^. This situation integrates the delineation of the Nursing profession in Brazilian lands and centuries-old Portuguese lands. In both experiences, nursing emerges as a discipline at the end of the 19th century, when the nursing occupation determines the object of study and there is an epistemological rupture, characterizing a status to knowledge as a profession^(2)^, thus giving Nursing the status of knowledge and scientific practice.

The social-historical and cultural framework of nursing organization has been investigated, discussed, and thought about since its creation. This is because it accompanies the care practices established in the domestic sphere, from the social imaginary, characterizing them in a science under construction for the daily search for qualifications and specifications that organize and sustain their actions, based on contextual changes since the 19th and 20th centuries, and also largely from the influences of scientific status in nascent development, as well as the definition of the doctrinal body of the occupation^(3)^. Moreover, in this scope, the Sociology of Professions within the reach of status in the care and investigative field, in the management of material and immaterial care in public and private treatment institutions at the hospital level, and in primary health care, reiterates the need for the specificities of the professional body. This, with a view to the autonomy and legitimacy of nursing for health care^(4,5)^.

Faced with this rhetoric, Eliot Freidson^(6)^ presents credentialism, expertise, and autonomy as the basis for an occupation to be recognized as a profession. However, the sociologist’s first analysis comes from fundamental characteristics for achieving the aforementioned, qualification through prolonged and in-depth training, and the idea that skills are aimed at the collective, at society. This highlights the nurse’s ability to develop care within ethical and legal standards^(7)^, strengthening autonomy and, thus, the recognition of the need for this professional in the health construct of society. It is at this moment, then, that Nursing has defined its professional status. Paying attention to the entire dialogue, now presented to the development of the profession, specialized knowledge incorporates professional autonomy through the relationship and enhancement of political articulation, credentialism of professional disciplining bodies, and ongoing development of knowledge improvement^(4)^. In this context, improvements in nursing knowledge and competence must be combined with the professionalism of the members who develop Nursing.

Professional autonomy is endorsed by the competence to provide health care with relative independence. In the Freidsonian view, autonomy is based on qualification credentials for work and specific knowledge that guarantee the diagnosis of the health status, not limited to the diagnosis of the disease^(6)^. From this perspective, Nursing, as a profession in the health sector, highlights, based on credentialing by representative entities and the legal formality of the State, as well as the social recognition of the profession, specificities that consolidate it as an academic and consultation profession^(4)^.

This defines, in this study, the concept of autonomy, which deals with authority over the practices and knowledge of care, related to the auspices of the profession. It determines its own educational standards, legal accreditation, and developed by professional members, and its practice is not subject to evaluation by lay people^(6)^, all reiterated by the credentialism represented by the organizational and disciplinary entities of the profession and formal education with specificities restricted to the professional group.

Professional autonomy is intertwined with variables that identify authority within the scope of health work. It is defined by professional-patient, professional-institution, professional-professional relationships in the health area, thus evidencing two levels of authority according to Freidson, an authority based on expertise/theory and a socioeconomic authority^(6,8)^. The authority of expertise establishes control over what is specific to the profession, the essentials, its work, technique, and specific thoughts. Socioeconomic autonomy is linked to the ability to organize and govern work, behavior and relationships between professional members and other members of society.

The authority required for the profession is declared by Freidson as the ability to control and evaluate the work carried out by members of the profession^(6)^. This translates into an ideal of service that makes technical/theoretical activity a necessity and meets the social interests, those of the community, and not specifically of the professional.

From the perspective of bringing to discussion the autonomy within the Portuguese and Brazilian Nursing legislation, it is observed that it meets the sociology by Eliot Freidson, in which Nursing shows itself as a consultation profession, based on esoteric knowledge through systematizations of caring, educating, managing, and researching. This encourages us to reflect analytically on the autonomy of nursing, as indicated by Bellaguarda et al.^(4)^, as a science, art, professional discipline, with a level of coverage similar to that of other professions in the health field. Therefore, the question arises: What are the convergences of professional autonomy expressed in Brazilian and Portuguese legislation for the Nursing profession? This question justifies this revisit to the professional autonomy of nurses, revealing identity roles and the visibility of the health profession in the countries under study. From this perspective, the objective of the study is to analyze the convergence of nurse’s autonomy expressed in Brazilian and Portuguese professional practice legislation, from the perspective of Eliot Freidson’s Sociology of Professions.

## METHOD

### Design of Study

To organize this study, a protocol was initially created with the constituent elements: demarcation of the theme, objective of the study, guiding question and research modality, documentary sources and strategies to be implemented for searching for data, internal and external criticism of the documents, and data synthesis. Thus, a qualitative study was carried out, with a documentary approach, from a social-historical perspective, carried out between two professional realities, within the space-time period from 1986 to 2022, justified by covering Brazilian and Portuguese Laws and standards, considering the initial date by Law no. 7498/1986^(9)^ of the Professional Practice of Nursing in Brazil and the limit is the year 2022 by the last change in this Law, to insert the minimum wage for Nursing, with Law no. 14.434/2022^(10)^. Composing this time frame are the Portuguese legislation regulating the professional practice of nursing by Law no. 161/1996^(11)^ or Regulation of the Professional Practice of Nurses (REPE) and Regulation No. 613/2022^(12)^, which defines the Nurses Act. Legislation, decree-laws, regulations, and curricular guidelines for nursing education were included electronically and officially disseminated in the profession’s disciplinary agencies in both countries, within the space-time framework.

### Study Context

Study carried out based on the knowledge network established between two Higher Education Institutions, a Brazilian one and a Portuguese one, to study the post-doctoral path and social-historical interest. The Brazilian educational institution is located in the south of the country, in the State of Santa Catarina, based on the Nursing School created 53 years ago in 1969 and the Postgraduate Program in Nursing 45 years ago, in 1977. The Portuguese nursing school, located in the center of Portugal, in the city of Coimbra, is the result of the merger in 2006 of two schools, one founded in 1881 and the other in 1971, located in the university city that is a world heritage site of the United Nations Educational, Scientific, and Cultural Organization, which since 2002 has been developing high-level research in Nursing Knowledge at the Health Sciences Research Unit.

This research, based on the existing partnership between the schools, allowed the conduction of a documentary study, on the digital platforms of the Nursing Council and Order of Brazil and Portugal, on the digital platform of the Federal Nursing Council (COFEN) and the Regional Council of Santa Catarina (COREN-SC), as well as on the page online of the Brazilian Ministries of Health and Education. In Portugal, the study was based on free, public, and virtual access documentation from the Order of Nurses and official websites of the Portuguese Government, electronic *Diário da República*, Ministry of Health and Ministry of Science, Technology, and Higher Education.

### Documentary Sources

The corpus of this study consisted of documentary analysis carried out in the social-historical records of legislation and normalization of professional standards, established by the disciplinary and regulatory agencies of Nursing in Brazil and Portugal, even though the professionalism of nurses from both realities is asymmetrical, as evidenced in the legislations, since in the Portuguese reality Nursing is undertaken only by the Nurse. Specifically, in Brazil, nursing is characterized by a partial division of work into Nurse, Nursing Technician, and Nursing Assistant. The discussion in this study is centered on the nurses’ potential autonomy and their specific competence, which implies a textual analysis of legislation.

The selected materials were organized according to the selection criteria and characterized in Brazilian and Portuguese documentary sources in type of document, year of creation, and provision of the standard (Chart 1).

**Chart 1:** Documentary sources of the study - Coimbra, Portugal, 2022.

Brazilian Documentary Source			Portuguese Documentary Source		
Document Type	Year of creation	Standard provision	Document Type	Year of creation	Standard provision
1. Professional Nursing Practice Law no. 7.498	June 25, 1986	Provides for the regulation of the practice of Nursing and provides other measures.	1. Regulation of the Professional Practice of Nurses (REPE) no. 161	September 4, 1996	It defines the general principles regarding the professional practice of nurses, constituting the Regulations for Nurses’ Professional Practice (*REPE*).
2. Resolution no. 358	October 15, 2009	Provides for the Systematization of Nursing Care and the implementation of the Nursing Process (NP) in public or private environments, in which professional Nursing care occurs, and provides other measures.	2. Decree-Law no. 104	April 21, 1998	Referência legal fundadora do escopo jurídico referenciador da profissão.
3. COFEN Resolution no. 564 Federal Nursing Council (COFEN)	November 6, 2017	Approves the new Code of Ethics for Nursing Professionals, as per the annex to this Resolution, for the observance and respect by Nursing professionals.	3. Law no. 156	September 16, 2015	Statute of the Order of Nurses, approved by Decree-Law no. 104/1998, amended and republished on the Law establishing the legal regime for the creation, organization, and operation of professional public associations.
4. CNE/CES Resolution nº 3 National Education Council (CNE) Chamber of Higher Education (CSE)	November 7, 2001	Establishes National Curriculum Guidelines for the Undergraduate Nursing Course.	4. Decree-Law no. 353	September 3, 1999	Approves an integrated plan of structuring measures for the development of human resources in the field of health that, in the nursing domain, is general training in undergraduate teaching courses and in graduate specialization courses.
5. Law no. 14.434	August 4, 2022	Amends Law No. 7.498, of June 25, 1986, to establish the national wage floor for Nurses, Nursing Technicians, Nursing Assistants, and Midwives.	5. Regulation No. 613/2002	July 8, 2022	Regulation that defines the nurse's act.

**Source**: From the authors, 2022.

### Selection Criteria

For the selection of documents, the inclusion criteria were observed: legal documents digitized in the time frame of this study, made available electronically in the professional nursing disciplinary agencies in both countries, and freely accessible. Exclusion criteria: specific documents of acts of nurses in the Covid-19 Pandemic.

### Data Collection and Organization

Data were collected between September 14 and October 31, 2022, over a period of two hours a day, in the afternoon shift, on two days of the week, for six weeks, totaling 24 hours. The start date of the search and data collection coincides with the beginning of the post-doctoral journey of one of the authors in Portugal. The closure took place at the expense of the documents necessary for the study.

A search was carried out on the digital platform of the profession’s organizational entities in both countries, laws and resolutions dealing with the rules of professional practice, to approximate the characteristics of a profession described by Eliot Freidson: autonomy, credentialism, expertise^(6)^.

In the initial search, 12 documents were found that corresponded to the inclusive criteria and rationale for nurses’ professional autonomy, 10 of which were used for data extraction and analysis. Two documents were excluded because they contained generalized data regarding autonomy.

Therefore, 5 documents referring to Brazilian legislation and 5 to Portuguese legislation were selected, being organized in a table of *Word* 2010 containing: type, year, and provision of professional standardization, articles and textual sources that bring archetypes of autonomy in the areas of professional practice, expertise, and qualification credentials of this autonomy expressed in legislation.

### Data Analysis

In the analysis, the treatment of materials followed social-historical research, the pre-analysis phase, observing the rules of exhaustiveness, representativeness, homogeneity and relevance in the selection of documents to be investigated. The second stage of the process dealt with mining, listing the specific legislation for the professional practice of nursing, and the respective education and ethical documents relating to the profession. In exploring the material, the documents were identified, a thorough reading was and later an in-depth reading were done, observing the presence of the search term “autonomy” in the articles of legislation and proceeding to the stage of exploration, through a synthesis chart, referring to the extraction of content, coded and categorized to continue the processing phase of the results obtained and interpretations. In this last stage, the social-historical writing of the nurses’ autonomy took place, congruent with the laws of professional nursing practice in Brazil and Portugal.

The convergence of professional autonomy in Portuguese and Brazilian Nursing practice legislation addresses, in this study, three thematic coding blocks: education/training/academy, which reflects ethics, esoteric knowledge, professional identity, and professionalism of the members. In the second composition, assistance/consultation/care/contexts, issues of ethics, nursing practice, and clinical nursing practice and the Systematization of Nursing Care (*SAE*) emerged. Finally, in the third thematic block credentialism/management, ethics, self-regulation, work organization and professional councils, orders and associations were highlighted. The convergence was achieved through interpretation based on the contextualization of the records in legislation in the reality of each country and Freidson’s sociology of professions.

### Ethical Aspects

The documents analyzed are in the public domain and were not submitted to or assessed by the Research Ethics Committee. They followed the characteristics required to carry out documentary research, organicity, being produced due to the functions of the entity representing the profession, uniqueness, reliability, and authenticity due to the veracity of the specific legislative document for the normalization of professional standards^(13)^. Study guided by *Consolidated Criteria for Reporting Qualitative Research* (COREQ) for the description and methodological composition^(14)^.

## RESULTS

Nursing professional rules in Brazil are set out in two Laws with specificity on the practice of Nursing, disciplinary issues, and professional organization. Higher-level nursing education corresponds to the regulations established by the Ministry of Education within the national curricular guidelines.

In Portugal, standardization occurs in the Decree-Law on professional disciplining and organization and provides for the Order of Nurses and Professional Public Associations, with legislation pertinent to health training and especially in nursing, and through the Portuguese Ministry of Education.

The legislation is presented (Chart 1), in which the themes of autonomy and autonomy of the Limits of the Multiprofessional Relationship were analyzed.

To analyze this convergence of autonomy in legislation, the categories were listed, based on thematic blocks: Autonomy of knowledge and competence specific to the profession; Autonomy in disciplining: from training to professional practice.

## DISCUSSION

Autonomy of knowledge and competence specific to the profession.

Although Portuguese secular history is remarkable, with regard to the organization and credentialism of the Nursing profession, it happens a little later than in Brazil, 1996 and 1986 sequentially. Professional standards in both countries have a similar structure, complying with the rules on rights and duties and deontological ethics^(8,10)^. From this perspective, the discussion is presented in the three dimensions achieved in the analysis to address the convergences in legislation and the aspects of understanding the nurses’ autonomy in realities, showing nursing as a consultation practice and not just a task performer.

The autonomy presented in the standardization of professional nursing practice in Brazil and Portugal, within the Freidsonian perspective, has a different understanding. This is because standardizations highlight a strong normalizing theoretical focus in Portuguese legislation and, in Brazilian legislation, they have less intense highlights in terms of the frequency of focus on autonomy in legislative articles.

It is observed, in Brazilian documents, that the reference to autonomy appears in some articles of legislation that respect the independence of the person served by the nurse^(9,15–17)^. In Portugal, autonomy is found in most standardization articles, which cover nurses, other professionals, and people cared for^(11,12,17)^.

It should be noted that the inclusion of the term autonomy does not necessarily have a connotation of independence or authority in health care. According to the qualifying characteristics of a listed occupation^(6)^, professional autonomy is not absolute when referring to professions in the health sector, exhibiting a certain inconsistency when referring to medicine. Freidson^(6)^ also characterizes Medicine as a profession with power over others in the health sector. Undoubtedly, in the history of health, the work process in medicine has always highlighted imperative autonomy. However, professions are interdependent and there is no absolutism in one occupation or another.

Consequently, this proposal is an analysis of the interior of the Nursing profession and the autonomy of this practice alone and its credentials, judging the object of study, care, and the object of work, the person^(18)^. Thus, autonomy in Freidson, for nursing, is not absolute and focuses on the non-reciprocity of care power between doctor and nurse and vice versa. The physician is brought up by Freidson as a professional superior to other professions, as a model. Moreover, in the field of nurse activity, these will not reach the status of the physician^(6)^.

Based on this position, the discussion of the Nursing profession is pertinent. It is not the status of the Physician that Nursing demands, but the status of authority over their work and practice. This as an autonomous profession within its own expertise and credentials, for health care and from a perspective of professional identity for society.

Independence of care, teaching, research, extension, management and health services has already been established within the scope of nursing. Furthermore, it has authority supported by normalizations and the need for this to be done by society. In analysis, the dimensions of the specific autonomy of the profession, ethics within the limits of the multi-professional relationship, and training for professional practice are presented^(6)^.

In this approach, autonomy in the ethical limits of the multi-professional relationship consolidates knowledge as a fundamental value for the action of social agents. This knowledge is built gradually, and with strong historical and cultural adherence, and changes, from this perspective, need solid foundations to be transformed, covering other issues within the scope of the labor, economic and administrative market in each profession^(19)^, having a cultural implication that is defined by the health need in each country. It refers to the job market, financial interest and fair and equitable salary recognition, which must come together to valuing and enhancement of professional work^(10)^, in which the struggles in favor of this finding characterize the control of the profession in both countries.

In Brazil, in legislation, issues of professional discipline are under the auspices of the Federal Nursing Council (COFEN) and the Regional Councils (CORENs), which defend and discipline the profession, with regard to professional practice, assistance, and management^(15)^. Training and education requirements are addressed; however, the councils do not have disciplining and direct action on the nurses’ training process. However, professional practice comes from qualified and higher education; therefore, to this end, requires regulations that are arranged by the Ministry of Education in accordance with the credentialism for professional practice ordered by the professional council^(20)^.

In Portugal, the Ministry of Education, by Decree Law 1999, implements the training regulations for Higher Nursing Schools and brings legislation to ensure the essential elements for their development: autonomy and independence among health professions, non-functional dependence on nurses to other professions and respect for technical autonomy and their own skills^(1,8,19)^. These standards guarantee professional relations, organization and health work in both countries.

In the sociology of professions supported by Freidson^(6)^, knowledge underlies, together with qualification credentials, the autonomy of the professional members in question. Thus, in the analysis of autonomy, within Nursing standardization in Brazil and Portugal, similarities are expected, which leads to the research of particularities in one and another situation.

In the Portuguese legislation, the nurse is someone qualified from a formal and higher nursing course that gives him/her scientific, technical and human competence^(12,21)^. The initial concept is directly linked to knowledge, training for care management with a recognized professional title. The concept of nurse in Brazilian standards previously highlights autonomy according to ethics followed by technical-scientific and theoretical-philosophical knowledge^(9,20)^. The regulations state that knowledge is the basis for professional identity and that it is from knowledge that the professional body has the qualifications to achieve autonomy.

Institutions of higher nursing training in the role of professional legislation present themselves as indicators of solutions and development of the own knowledge specific to the category, for respectability and recognition by the State and Society^(6,16)^. In this scope, the esoteric knowledge brought by Freidson^(6)^ is evident; the specific knowledge of the profession has to be well established, which, we understand, guarantees legitimacy to the professional group through knowledge that is strong, of results (evidence), substantiated (scientific), intrinsic (proper), and distinctive (attitudinal and skills).

### Autonomy in Disciplining: From Training to Professional Practice

It should be noted that professional autonomy is consolidated through knowledge in the legislation of both countries. There are studies showing that the greatest professional satisfaction lies in the autonomy acquired and that it is a factor for maintaining the nurse’s work in a given institution^(22,23)^.

The Systematization of Nursing Care (*SAE*) appears as esoteric knowledge in the Brazilian legislation, with intense normalization by the Nursing Process (NP), the theoretical-methodological and philosophical references that add to the health work process. In Portugal, the skills and attitudes for specific nursing knowledge are visible and evidenced in legislation as functions under ethics and respect for citizens’ rights and functional complementarity actions with other health professionals^(9,23,24)^.

The autonomy and specific competencies of the Nursing profession in the revisited legislation refer to independence in the development of its functions related to teaching, research, extension and management, covering specificities in the training of future nurses, in nursing research, in caring for people and in the management of nursing and health services. The authority that expresses the theoretical autonomy understood by Freidson is described in the standardizations, and appears to be coherent with the specific knowledge of this profession^(6)^, which historically records in the regulations the evolution of recognition by the state of this knowledge of nurses for the action and authority of their practice. Therefore, the level of authority linked to expertise is defined by this knowledge, but will only be leveraged by the professional members’ appropriation of the specific body of this knowledge and the position they themselves determine in the professional-professional and professional-society relationships.

Brazilian regulations bring the specificity of SAE and NP, detailing this esoteric knowledge and the autonomy it grants to nurses, since autonomy and authority in documents is particularized as an exclusive activity of this professional^(4,25)^, as there are other categories structuring the profession in Brazil. Portugal presents nurse training and there is no category of Nursing Assistant and Nursing Technician. All care activities are carried out by nurses. This characteristic of the professionalism of nursing members in the countries in question also influences how this autonomy and authority possibly occurs.

We discuss what the legislative documents state about the issue of autonomy for nursing training to be reflected in the provisions, when they frame the limits and educational and training possibilities for professional practice. However, professional autonomy/independence has to be understood as the ability of professional members to control time and work practices^(6,26)^.

It alerts to the deepening of the professionalism of the members who make up the nursing workforce. This control over the work is what reflects autonomy, and refers to interprofessionality and the imperative credentialism over professional acts. Thus, it covers the organization of competences that prepare nurses for autonomous practice, within their context of expertise with certain independence.

In this scope, grounded knowledge is directly close to credentialism, which guides discipline based on knowledge, for professional practice^(22)^. Professional independence is consolidated through the knowledge and evolution of nursing as a discipline, which is historically organized based on theoretical-methodological references in the organization of work, teaching, research, and health management itself. According to Pires^(18)^, nursing, as a discipline, is one that, defining the object of study and care under theoretical and methodological strategies, presents convergence with paradigms. It is normal that the organization and construction of a discipline does not develop without political, economic and social disputes and interests^(18)^. As a result, knowledge is the fundamental value for the action of social agents. Knowledge is the basis for professional organization, for the description and development of rules, qualification credentials, and standards for ethical coexistence and limits of professional competence^(4,6,15,27)^.

These standardizations guarantee professional relations, organization and health work in both countries, in a complex confluence according to Brock and Saks^(28)^, since approaches to knowledge qualification and research, within the scope of professions, direct towards domain management and professional control.

The professional nursing supervision and disciplining agencies in Brazil and Portugal are responsible for representing and defending the general interests of nursing care users and regulating the profession, which directly implies professional autonomy, incorporated into the nurses’ competence to take decisions^(28)^. This is observed in the legislation analyzed, in which training, ethical precepts and professional disciplinarians maintain a dialogue among the management of health services, professional order and council, professional members, and training institutions^(29,30)^.

This organizational capacity defines the need for the profession in the health sector for society, characterizing a social authority, which depends on the professional category and legislative agencies to consolidate expertise, disciplining, and credentialism, in addition to a political, economic and cultural dependence with the State’s approval.

### Study Limitations

The limitations to the completeness of the study focus on the multiplicity of standardizations of professional practice and, mainly, on the fact that it is only a documentary analysis, which does not detail and deepen the autonomy developed in the daily work, education and management of nurses in both countries. However, documentary sources provide the opportunity for congruence of professional autonomy in light of the sociological references of professions.

### Contributions to the Field of Nursing

The documentary methodology and sociological analysis in the light of Eliot Freidson on the normalization of professional standards in Nursing in two different realities, in Brazil and in Portugal, stand out in this research. It takes analytical perspectives on professional practice legislation, allowing the monitoring of developments in the world of work and employability in updating credentialism, autonomy and expertise in professionalization policies in Nursing.

## CONCLUSION

The analysis shows that the interest in this discussion emerges for a visibility of the profession, that professional autonomy, based on the foundation of Freidson’s Sociology of the Profession, and the implications for the health of society refer to access to health, opportunities and availability of care to society, thus guaranteeing the nurses’ autonomy under interprofessional practices that are understood, ethical, and accessible to this society.

It should be noted that the autonomy set out in legislation must be analyzed from an ethical perspective, from the functionality of clinical nursing, and that it is necessary to understand what relevance this credentialism brings to this effectively autonomous and interdependent practice. It is considered that professional autonomy only becomes effective as the professionalism of professional members is established as such and that it is involved in ensuring accessibility to health for society. In the social-historical context of the organization of Nursing, both in Brazil and in Portugal, nurses’ autonomy follows an itinerary linked to educational/training/investigative improvement and health care for the community.

It is suggested that studies be developed that establish a relationship between professional autonomy expressed in legislation and the effectiveness of this independence and control in assistance and research and management applied in health services by nurses.
